# Introducing dental trauma (a non‐curricular topic) into medical education: A viewpoint

**DOI:** 10.1111/edt.12782

**Published:** 2022-08-17

**Authors:** Thai Yeng

**Affiliations:** ^1^ Faculty of Medicine, Medical Education University of New South Wales Sydney New South Wales Australia

**Keywords:** dental trauma, medical education, medical students, traumatic dental injury

## Abstract

The aim of this paper is focused on expanding medical students' knowledge and understanding of a non‐core curricular subject not normally offered in the medical curriculum. The critical juncture explored how to introduce a health‐related subject into medical education that complements without competing for space/time in medical students' core studies. Using dental trauma as an example, this paper supports the opportunity for medical students to learn online about managing dental injuries and to raise their awareness of the need for accurate diagnosis and treatment of traumatic dental injuries before they graduate as medical doctors.

## 1 INTRODUCTION

From a medical education perspective, developing an online learning platform to teach medical students a less prioritised, yet important, health‐related subject that is not currently offered at the medical school is essential for student learning. Online learning allows students to self‐learn anywhere, at any time.^1^ Providing an easy to access online course will help make online education more enjoyable and effective.^2^ As educators are regarded as learning facilitators, it is their role to create an environment that makes students' access to learning materials as uncomplicated and acceptable as possible.^3^ Applying the subject of dental trauma to showcase how to propose to the medical school curriculum committee that an online course could be used to introduce a new subject,^4^ and to develop an online learning course that expands knowledge on the management of dental injuries into medical education^5^ without amending or affecting the existing medical curriculum will benefit future medical doctors in general medical practice or specialties that assess and manage trauma.

The underlying premise of introducing a non‐curricular topic into medical education was that if learning the topic of dental trauma is presented as an interesting and important topic using an online course approach,^6^ and medical students are provided with adequate online learning resources through a facilitatory environment,^7^ then students may be more motivated to learn about traumatic dental injuries (TDIs).^8^ In the absence of a dental educator at the medical school, offering online learning becomes an ideal and practical solution to provide medical students with the knowledge to assess and manage TDIs.^6^ Overall, the aim of the online course is to equip medical students with basic dental knowledge to accurately diagnose and provide appropriate emergency management for TDIs,^9^ plus how to communicate effectively with dentists and dental specialists regarding the extent and nature of TDIs^4^ to ensure greater continuity and consistency of the patient's management plan.

In creating a framework for developing online learning platforms for medical students, medical educators will be able to introduce the topic of dental trauma by encouraging self‐learning outside the formal medical timetable.^10^ Online learning would first present theoretical foundations for the new subject^11^ and then allow medical students the opportunity for further clinical training to supplement the new knowledge gained.

## 2 CONCLUSION

The overarching aim was to find an alternative way to expand medical students' theoretical knowledge of dental trauma, a non‐core curricular topic, by introducing it into medical education without occupying formal teaching hours. The proposal to use an online dental trauma course provided a pathway for cross‐disciplinary learning in medical education.

## FUNDING INFORMATION

Australian Government Research Training Program (RTP) Scholarship.

## CONFLICT OF INTEREST

No potential conflict of interest relevant to this article was reported.

## Data Availability

The data that support the findings of this study are available from the corresponding author upon reasonable request.
